# Impact of cumulative cisplatin dose in childhood nasopharyngeal carcinoma based on neoadjuvant chemotherapy response in the intensity-modulated radiotherapy era: a real-world study

**DOI:** 10.1186/s12935-021-02281-4

**Published:** 2021-11-12

**Authors:** Ya-Nan Jin, Meng-Yun Qiang, Meng-Meng Liu, Zhi-Bin Cheng, Wang-Jian Zhang, Ian Ryan, Tia Marks, Ji-Jin Yao, Liang-Ping Xia

**Affiliations:** 1 VIP Region, State Key Laboratory of Oncology in South China, Guangdong Key Laboratory of Nasopharyngeal Carcinoma Diagnosis and Therapy, Collaborative Innovation Center for Cancer Medicine, Sun Yat-sen University Cancer Center, Guangzhou, 510060 Guangdong China; 2 Department of Head and Neck Radiotherapy, The Cancer Hospital of the University of Chinese Academy of Sciences (Zhejiang Cancer Hospital), Institute of Basic Medicine and Cancer (IBMC), Chinese Academy of Sciences, Hangzhou, 310022 Zhejiang China; 3 Melanoma and Sarcoma Medical Oncology Unit, State Key Laboratory of Oncology in South China, Collaborative Innovation Center for Cancer Medicine, Sun Yat-sen University Cancer Center, 651 Dongfeng Road East, Guangzhou, 510060 China; 4 The Cancer Center of the Fifth Affiliated Hospital of Sun Yat-Sen University, Guangdong Provincial Key Laboratory of Biomedical Imaging, Zhuhai, 519000 Guangdong China; 5 Department of Medical Statistics, School of Public Health, Sun Yat-sen University, Guangzhou, 510080 Guangdong China; 6 Department of Environmental Health Sciences, School of Public Health, University at Albany, State University of New York, Rensselaer, 12144 USA

**Keywords:** Nasopharyngeal carcinoma, Cumulative cisplatin dose, Children and adolescents, Tumor response, Neoadjuvant chemotherapy

## Abstract

**Background:**

We aimed to comprehensively investigate the optimal cumulative cisplatin dose during concurrent chemoradiotherapy (CC-CCD) for locoregionally advanced nasopharyngeal carcinoma (CA-LANPC) with different tumor responses after neoadjuvant chemotherapy (NAC).

**Methods:**

Patients with CA-LANPC who underwent NAC followed by cisplatin-based concurrent chemoradiotherapy were retrospectively analyzed. Evaluation of tumor response in patients was conducted by Response Evaluation Criteria for Solid Tumor (RECIST) 1.1 after two to four cycles NAC. Multivariate Cox proportional hazards models were used for prognosis. Recursive partitioning analysis (RPA) was conducted to classify participates and predict disease-free survival (DFS).

**Results:**

One hundred and thirty-two patients with favorable response after NAC were included. The median CC-CCD was 163 mg/m^2^ (IQR, 145–194 mg/m^2^), and 160 mg/m^2^ was selected as the cutoff point to group patients into low and high CC-CCD groups (< 160 vs. ≥ 160 mg/m^2^). There was significant improvement in 5-year DFS (91.2% vs. 72.6%; P = 0.003) for patients receiving high CC-CCD compared to those receiving low CC-CCD. Multivariate analysis revealed that CC-CCD, T stage, and Epstein–Barr virus (EBV) DNA were independent prognostic factors for DFS (P < 0.05 for all). Patients were further categorized into two prognostic groups by RPA: the low-risk group (T1-3 disease with regardless of EBV DNA, and T4 disease with EBV DNA < 4000 copy/mL), and the high-risk group (T4 disease with EBV DNA ≥ 4000 copy/mL). Significant 5-year DFS improvement was observed for the high-risk group (P = 0.004) with high CC-CCD. However, DFS improvement was relatively insignificant in the low-risk group (P = 0.073).

**Conclusions:**

CC-CCD was a positive prognostic factor for responders after NAC in CA-LANPC. Furthermore, CC-CCD ≥ 160 mg/m^2^ could significantly improve DFS in the high-risk group with CA-LANPC, but the benefit of high CC-CCD in the low-risk group needs further study.

**Supplementary Information:**

The online version contains supplementary material available at 10.1186/s12935-021-02281-4.

## Background

In children and adolescents (age ≤ 18 years), nasopharyngeal carcinoma (NPC) is rare and accounts for only 1–2% of all NPC cases [1, 2]. Given the rarity of childhood NPC and the difficulty of conducting relevant prospective studies, treatment of childhood NPC traditionally follows guidelines applied adults. The National Comprehensive Cancer Network (NCCN) Guidelines recommended neoadjuvant chemotherapy (NAC) followed by concurrent chemoradiotherapy (CCRT) as level 2 A evidence for locoregionally advanced NPC (LANPC) [3]. At present, the cumulative cisplatin dose during concurrent chemoradiotherapy (CC-CCD) has been considered an important treatment for LANPC. However, the optimal cutoff value of CC-CCD in NPC remains controversial. The study by Peng et al. [4] showed 200 mg/m^2^ CC-CCD may suffice in achieving survival benefits. While Lv et al. found 160 mg/m^2^ CC-CCD may be sufficient to precipitate beneficial antitumor effects after NAC [5]. Of note, these findings were mainly limited to adult patients (age > 18 years), while patients aged 18 years and younger were excluded from these studies [4, 5]. Compared to adult patients, tolerance and sensitivity to chemotherapy in children and adolescents with LANPC (CA-LANPC) are markedly different [6, 7], and the optimal value of CC-CCD for CA-LANPC remains unclear.

NAC, given before CCRT, has been widely proven to be a feasible neoadjuvant treatment with satisfactory efficacy in LANPC during the past decade [8–10]. However, patients treated with the same NAC regimen can still show substantial heterogeneity in tumor response. Generally, patients who achieved complete response (CR)/partial response (PR) after two to four cycles of NAC were more likely to have increased sensitivity to NAC compared to those who suffered stable disease (SD)/disease progression (PD). Consequently, patients who suffered SD/PD after NAC had inferior locoregional relapse-free survival (LRFS) and disease-free survival (DFS) compared to patients reaching CR/PR after NAC [11, 12]. We therefore speculate that the optimal CC-CCD is likely distinct for patients with varied NAC responses. However, there is a lack of systematic investigation to evaluate the optimal CC-CCD in CA-LANPC with varied NAC response.

Given this evidence, we conducted a real-world research on CA-LANPC to elucidate the significance of CC-CCD in survival outcomes based on their tumor responses after two to four cycles NAC. Moreover, the therapeutic values of high or low CC-CCD were compared in various patient risk groups. Our results may provide oncologists better treatment strategies for CA-LANPC.

## Materials and methods

### Patients cohort

We retrospectively investigated the NPC-specific database which was provided and approved by the big-data platform at our center between September 2007 and April 2018. Eligibility criteria for our study included: (a) confirmed pathological NPC at III–IVB NPC (7th edition of the AJCC [American joint Committee on cancer] staging system [13]); (b) no other malignant disease; (c) age ≤ 18 years old at initial diagnosis; (d) first line NAC followed by cisplatin-based CCRT; (e) available imaging evaluation data after 2–4 cycles of NAC; (f) treated with intensity-modulated radiotherapy (IMRT); and (g) adequate organ function. A series of routine exams were conducted on each patient prior to treatment. Serum Epstein–Barr virus (EBV) DNA was quantitatively measured by polymerase chain reaction prior to treatment. This study received approval from the Research Ethics Committee at our institution. Patients informed consents were obtained for the entire cohort.

### Treatment and evaluation of NAC response

NAC followed by cisplatin-based CCRT was delivered to each patient. NAC regimens included PF [5-fluorouracil (800–1000 mg/m^2^, by 120-h continuous intravenous infusion) and cisplatin (80–100 mg/m^2^ on day 1)], TP [cisplatin (75 mg/m^2^ on day 1) and docetaxel (75 mg/m^2^ on day 1)], TPF [docetaxel (60 mg/m^2^ on day 1), cisplatin (60 mg/m^2^ on day 1), and 5-fluorouracil (500–600 mg/m^2^, by 120-h continuously intravenous infusion)], and GP [gemcitabine (1000 mg/m^2^ on day 1, 8) and cisplatin (80 mg/m^2^ on day 1)]. Two to four cycles NAC were administered at three-week intervals. Another MRI was conducted for all patients at 2 to 3 weeks after the final NAC cycle. Response Evaluation Criteria in Solid Tumors version 1.1 (RECIST 1.1) [14], which is widely used to evaluate the treatment of tumor response in various solid tumors [15, 16], was used to evaluate tumor response to NAC. In the current study, patients experiencing CR/PR were classified as favorable responders, and patients suffering PD/SD were classified as unfavorable responders.

IMRT was delivered to all patients on a conventional schedule (one fraction per day, five days per week). Prescribed doses for CA-LANPC complied with our center’s guidelines. In detail, prescribed doses of the planning tumor volume (PTV) for gross tumor volume (GTVnx) was 66–72 Gy; PTV of the involved cervical lymph nodes (GTVnd) was 64–70 Gy; PTV of high-risk clinical target volume (CTV1) was 60–64 Gy; and PTV for low-risk clinical target volume (CTV2) was 50–54 Gy in 30 to 33 fractions. The detailed introduction on IMRT planning was reported in our previous studies [17, 18]. Concurrent cisplatin-based chemotherapy was administered weekly (30–40 mg/m^2^ each week) or tri-weekly (80–100 mg/m^2^ every 3 weeks).

### Outcome and follow-up

DFS, given as the time from the NAC initiation to the date of disease progression or until any causes of death, was the primary endpoint. Other endpoints included overall survival (OS) (given as the time from the NAC initiation to the day of death), distant metastasis-free survival (DMFS) (given as the time from the NAC initiation to the day of distant metastasis), and LRFS (given as the time from the NAC initiation to the day of local/regional recurrence). Patients were monitored every 3 month during the first 2 years, every 6 months during the 3rd to 5th years, and once a year thereafter.

### Statistical analysis

Host factors [i.e. sex, age, smoking history, BMI (body mass index), and EBV DNA], tumor factors (i.e. T stage, N stage, and overall stage), and treatment factors [i.e. cumulative cisplatin dose during NAC (NAC-CCD), NAC cycles, NAC regimens, adjuvant chemotherapy, radiation dose, and CC-CCD] were all considered as covariates. Age, radiation dose, BMI, and NAC-CCD were transformed into categorical variables according to their median splits. As described in previous studies [19], patients were categorized into low- and high-EBV DNA groups based on the cutoff value of 4000 copies/mL. Since the median NAC-CCD was 181.8 mg/m^2^ (interquartile range [IQR], 141.7–221.1 mg/m^2^), 180 mg/m^2^ (< 180 vs. ≥ 180 mg/m^2^) was set as the cutoff point to classify patients into low- and high-NAC-CCD groups. Previously peer-reviewed data suggested a CC-CCD of 160 mg/m^2^ or 200 mg/m^2^ as necessary to confer survival for LANPC patients receiving NAC plus CCRT [4, 5, 20]. Therefore, we categorized patients into three groups based on CC-CCD (<160 mg/m^2^, 160–200 mg/m^2^, and > 200 mg/m^2^), and survival outcomes between different CC-CCD groups were compared. The clinicopathologic factors were compared using χ^2^ test for frequencies.

Cumulative survival rates were assessed using the Kaplan–Meier method and compared using a log-rank test. Multivariate Cox regression analysis confirmed the role of CC-CCD in DFS when adjusted for other possible factors. To attain the patients most likely to benefit from high CC-CCD, we performed individual comparisons of low- and high-CC-CCD for each risk group based on recursive partitioning analysis (RPA). The statistical significance was set as *P *< 0.05, and statistical tests were two-sided. All models were generated using R version 3.4.2 (http://www.r-project.org/).

## Results

### Patient characteristics

**Fig. 1:**
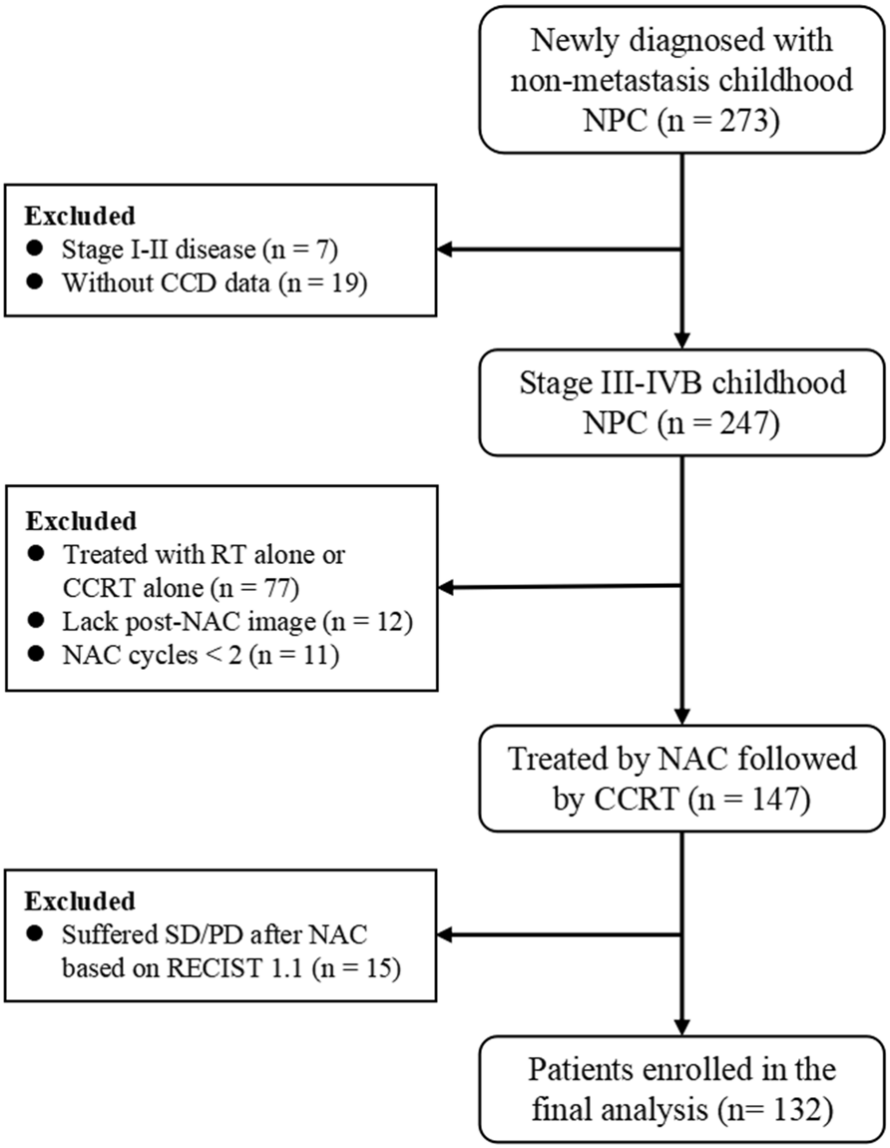
Flowchart of patient selection for the final analysis and exclusion criteria. *NPC* nasopharyngeal carcinoma, *CCD* cumulative cisplatin dose, *RT* radiotherapy, *CCRT* concurrent chemoradiotherapy, *NAC* neoadjuvant chemotherapy, *SD* stable disease, *PD* disease progression, *RECIST* Response Evaluation Criteria for Solid Tumor

Figure 1 presents the patient flow diagram. A total of 147 consecutive CA-LANPC patients who received NAC followed by CCRT were involved in this study. After two to four cycles of NAC, 15 patients (10%) suffered unfavorable responses (SD/PD), and 132 patients (90%) achieved favorable tumor response (CR/PR). The baseline characteristics of the 132 favorable responders were shown in Table 1. The percentages of patients grouped as stage III, IVA, and IVB were 27.3%, 36.4%, and 36.4%, respectively. A total of 60 (45%), 66 (50%) and 6 (5%) patients received two, three and four cycles NAC, respectively. The median radiation dose was 6800 cGy (IQR, 6600–7000 cGy). The median time to follow-up was 63.7 months (IQR, 40.7–88.9 months). During the follow-up, four patients (3%) exhibited locoregional recurrence, 17 (13%) developed distant metastases, and 14 (11%) died. For the entire group, the 5-year rates of DFS, DMFS, OS, and LRFS were 83.9% (95% CI 77.8–90.4%), 87.7% (95% CI 82.3–93.6%), 89.3% (95% CI 83.9–95.0%), and 97.5% (95% CI 94.9–100%) respectively.

**Table 1:** Baseline characteristics of study cohort (n = 132)

Characteristic	Entire cohort (n = 132)	CC-CCD, mg/m^2^		*P* value^a^
		< 160 (n = 52, %)	≥ 160 (n = 80, %)	
Sex				0.354
Male	92 (69.7)	39 (75.0)	53 (66.2)	
Female	40 (30.3)	13 (25.0)	27 (33.8)	
Age, years				0.494
≤ 15	69 (52.3)	25 (48.1)	44 (55.0)	
< 15	63 (47.7)	27 (51.9)	36 (45.0)	
Smoking history				0.170
No	123 (93.2)	46 (88.5)	77 (96.2)	
Yes	9 (6.8)	6 (11.5)	3 (3.8)	
BMI, m^2^				0.310
≤ 1.45	64 (48.5)	22 (42.3)	42 (52.5)	
> 1.45	68 (51.5)	30 (57.7)	38 (47.5)	
EBV DNA, copy/mL				0.293
< 4000	52 (39.4)	17 (32.7)	35 (43.7)	
≥ 4000	80 (60.6)	35 (67.3)	45 (56.3)	
T stage^b^				0.281
T1	1 (0.8)	1 (1.9)	0 (0)	
T2	4 (3.0)	1 (1.9)	3 (3.8)	
T3	59 (44.7)	27 (51.9)	32 (40.0)	
T4	68 (51.5)	23 (44.2)	45 (56.3)	
N stage^b^				0.165
N0	1 (0.8)	1 (1.9)	0 (0)	
N1	18 (13.6)	8 (15.4)	10 (12.5)	
N2	65 (49.2)	29 (55.8)	36 (45.0)	
N3	48 (36.4)	14 (26.9)	34 (42.5)	
Overall stage^b^				0.114
III	36 (27.3)	19 (36.5)	17 (21.3)	
IVA	48 (36.4)	19 (36.5)	29 (36.2)	
IVB	48 (36.4)	14 (26.9)	34 (42.5)	
NAC cycles				0.062
2	60 (45.5)	30 (57.7)	30 (37.5)	
3	66 (50.0)	21 (40.4)	45 (56.3)	
4	6 (4.5)	1 (1.9)	5 (6.2)	
NAC regimens				< 0.001
TPF	73 (55.3)	18 (34.6)	55 (68.8)	
TP	20 (15.2)	13 (25.0)	7 (8.8)	
PF	35 (26.5)	21 (40.4)	14 (17.5)	
GP	4 (3.0)	0 (0.00)	4 (5.0)	
Adjuvant chemotherapy				0.575
No	129 (97.7)	50 (96.1)	79 (98.7)	
Yes	3 (2.3)	2 (3.9)	1 (1.3)	
NAC-CCD, mg/m^2^				0.112
< 180	62 (47.0)	29 (55.8)	33 (41.3)	
≥ 180	70 (53.0)	23 (44.2)	47 (58.7)	
Radiation dose, cGy				0.386
< 6800	64 (49.6)	22 (44.0)	42 (53.2)	
≥ 6800	65 (50.4)	28 (56.0)	37 (46.8)	

*CC-CCD* cumulative cisplatin dose during concurrent chemoradiotherapy, *BMI* body mass index, *EBV* Epstein–Barr virus, *NAC* neoadjuvant chemotherapy, *TPF* cisplatin with 5-fluorouracil and toxoids, *TP* cisplatin with toxoids, *PF* cisplatin with 5-fluorouracil, *GP* cisplatin with gemcitabine, *NAC-CCD* cumulative cisplatin dose during neoadjuvant chemotherapy

^a^*P* values were calculated using the chi-square test or Fisher exact test if indicated

^b^According to the 7th edition of the Union for International Cancer Control/American Joint Committee on Cancer staging system

### Prognostic value of CC-CCD in favorable responders

The median CC-CCD to the favorable responders was 163 mg/m^2^ (IQR, 145–194 mg/m^2^), and the clinical characteristics were summarized in 40-mg intervals (Additional file 1: Table S1). Overall, 52 patients (39%) received CC-CCD < 160 mg/m^2^, 50 patients (69%) received a CC-CCD of 160–200 mg/m^2^, and 30 participants (22%) received CC-CCD > 200 mg/m^2^. No significant differences were noted with regard to sex, age, and TNM stage among each of the three groups (P > 0.05 for all; Additional file 1: Table S1). As shown in Additional file 2: Fig. S1, patients receiving CC-CCD of 160–200 mg/m^2^ exhibited significantly higher 5-year DFS rates than participants who received CC-CCD < 160 mg/m^2^ (94.0% vs. 72.6%, P = 0.010). Yet, no significant outcomes were seen in patients receiving CC-CCD > 200 mg/m^2^ and 160–200 mg/m^2^ for 5-year DFS (P = 0.261). Thus, we combined these above groups into one group with a CC-CCD ≥ 160 mg/m^2^. We selected 160 mg/m^2^ as the cut-off point to categorize patients into low- and high-CC-CCD group (< 160 mg/m^2^ vs. ≥ 160 mg/m^2^). The high CC-CCD group shared similar clinical characteristics with the low CC-CCD group except for NAC regimens, with those patients treated with NAC regimens of TPF were most likely to receive high CC-CCD (P < 0.001; Table 1).

The high CC-CCD group showed better 5-year DFS (91.2% vs. 72.6%; P = 0.003; Additional file 3: Fig. S2A) and 5-year DMFS (93.7% vs. 78.4%; P = 0.006; Additional file 3: Fig. S2B) than the low CC-CCD group. There was a distinct trend toward improved 5-year OS (94.8% vs. 81.7%; P = 0.071; Additional file 3: Fig. S2C) when comparing high CC-CCD group with low CC-CCD group. In contrast, there were no significant differences in the 5-year DFS (85.3% vs. 82.2%, P = 0.472; Additional file 4: Fig. S3A), DMFS (91.3% vs. 83.8%, P = 0.138; Additional file 4: Fig. S3B), OS (90.9% vs. 86.9%, P = 0.502; Additional file 4: Fig. S3C) and LRFS (96.9% vs. 98.2%, P = 0.406; Additional file 4: Fig. S3D) rates between high NAC-CCD group and low NAC-CCD group. In multivariate analysis, higher CC-CCD was an independent favorable prognostic factor for DFS (HR, 0.26; 95% CI 0.10–0.65; P = 0.004) and DMFS (HR, 0.28; 95% CI 0.10–0.81; P = 0.018). Apart from CC-CCD, T stage and EBV DNA were also found to be significantly associated with DFS and DMFS (P < 0.05 for all; Table 2). Although advanced T stage was significantly associated with poor OS (HR, 2.94; 95% CI 1.01–4.27; P = 0.046), there was only a borderline significance of EBV DNA for predicting OS (HR, 3.72; 95% CI 0.83–16.67; P = 0.057; Table 2).

**Table 2:** Multivariate analysis of prognostic factors for the entire group

Endpoint	Variable	HR	95% CI for HR	*P* value^a^
DFS	CC-CCD (< 160 vs. ≥ 160 mg/m^2^)	0.26	0.10–0.65	0.004
	T stage (T1–3 vs. T4)	2.66	1.07–6.62	0.036
	EBV DNA (< 4000 vs. ≥ 4000 copy/mL)	3.35	1.11–10.16	0.032
	Smoking history (no vs. yes)	2.53	0.72–8.92	0.148
DMFS	CC-CCD (< 160 vs. ≥ 160 mg/m^2^)	0.28	0.10–0.81	0.018
	T stage (T1–3 vs. T4)	2.34	1.03–5.62	0.041
	EBV DNA (< 4000 vs. ≥ 4000 copy/mL)	3.27	1.02–9.60	0.046
	Smoking history (no vs. yes)	3.50	0.97–12.64	0.056
OS	T stage (T1–3 vs. T4)	2.94	1.01–4.27	0.046
	EBV DNA (< 4000 vs. ≥ 4000 copy/mL)	3.72	0.83–16.67	0.057

*HR* hazards ratio, *95% CI* 95% confidence interval, *CC-CCD* cumulative cisplatin dose during concurrent chemoradiotherapy, *EBV* Epstein-Barr virus, *DFS* disease-free survival, *DMFS* distant metastasis-free survival, *OS* overall survival

^a^*P* values were calculated using an adjusted Cox proportional hazards model

### Role of CC-CCD based on RPA model

**Fig. 2:**
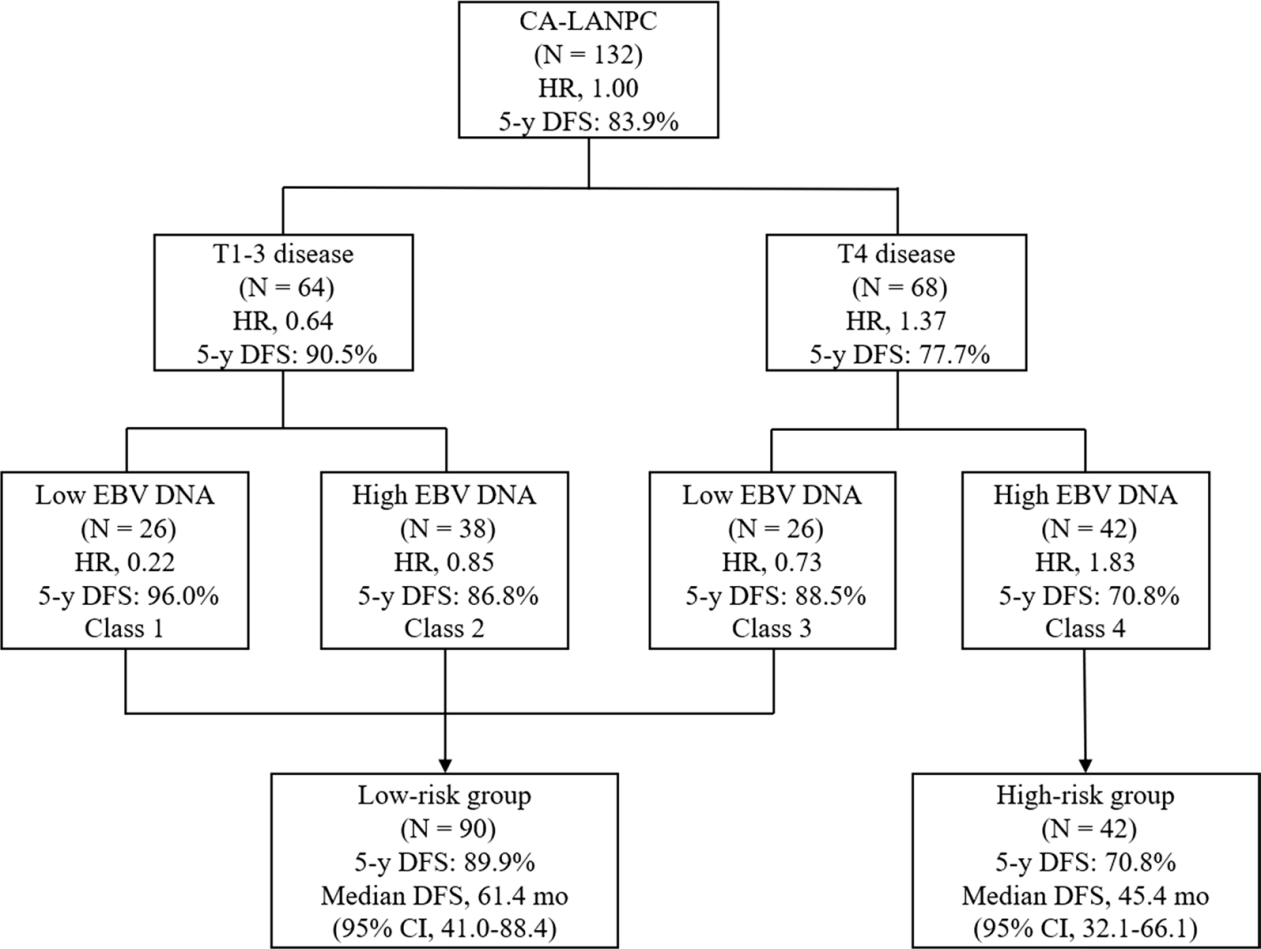
Prognostic grouping by recursive partitioning analysis in CA-LANPC for predicting DFS. *CA-LANPC* children and adolescents with locoregionally advanced nasopharyngeal carcinoma; *DFS* disease-free survival; *EBV* Epstein–Barr virus

**Fig. 3:**
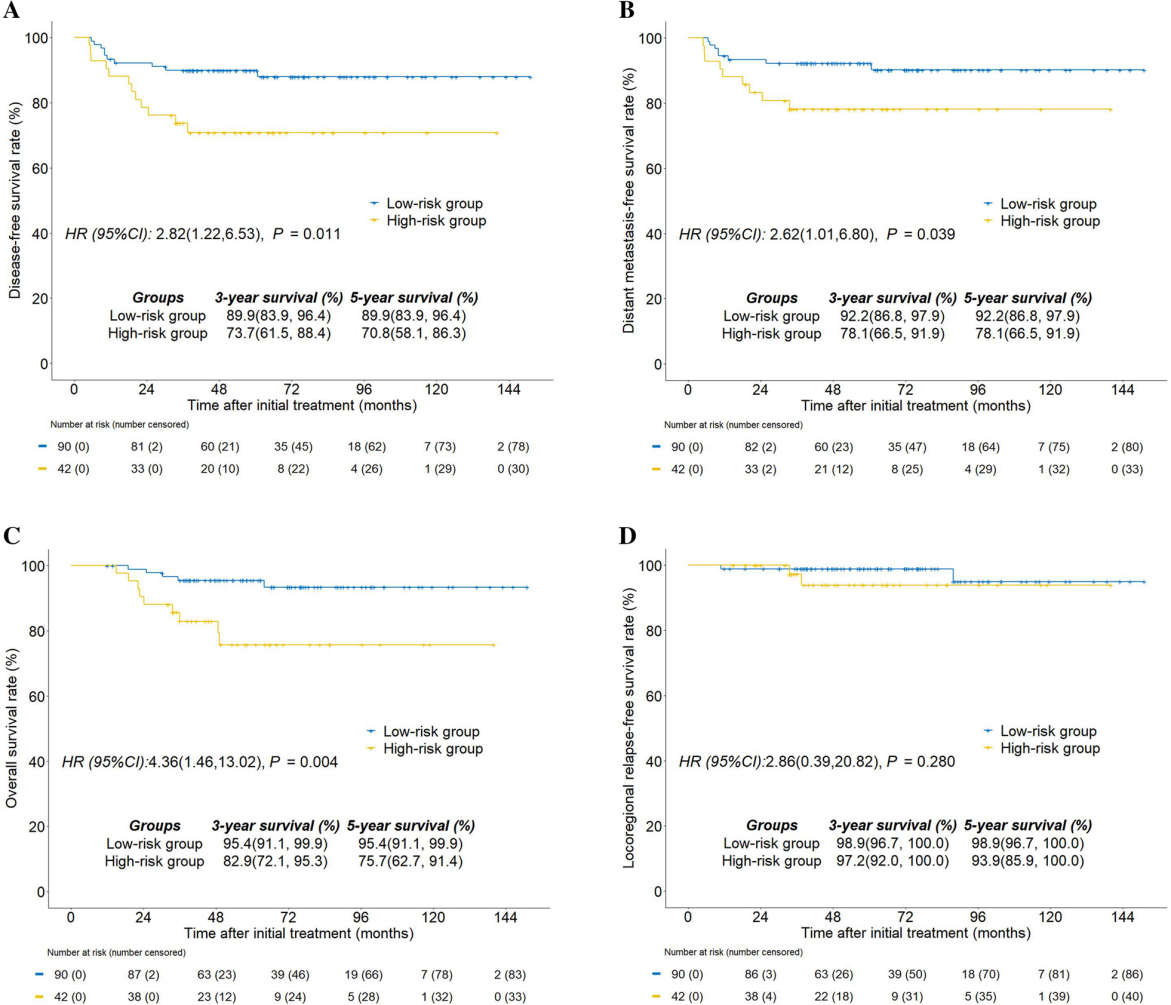
Kaplan–Meier survival curves for the high-risk group and low-risk group. **A** Disease-free survival, **B** distant metastasis-free survival, **C** overall survival, and **D** locoregional relapse-free survival high-risk group, T4 disease with EBV DNA ≥ 4000 copy/mL; low-risk group, T1–3 disease regardless of EBV DNA and T4 disease with EBV DNA < 4000 copy/mL

Patients were further categorized into variable risk groups based on T stage and EBV DNA given the DFS endpoint. As shown in Fig. 2, patients with CA-LANPC were segregated into 4 classes based on RPA. However, not all intergroup (e.g. class 1 to class 4) prognoses were significantly different (Additional file 5: Fig. S4). Patients from class 4 had the poorest DFS (5-year DFS, 70.8%) among the four groups and the DFS rates of class 1, 2, and 3 were comparable (class 1 vs. 2, P = 0.247; class 1 vs. 3, P = 0.346; class 2 vs. 3, P = 0.659; Additional file 5: Fig. S4), and therefore these three categories were merged. Consequently, our final RPA model categorized participants into two prognostic categories: low-risk group (T1–T3 with low or high EBV DNA, and T4 with low EBV DNA; n = 90) and high-risk group (T4 with high EBV DNA; n = 42), with corresponding 5-year DFS rates of 89.9% and 70.8%, respectively (P = 0.011; Fig. 3A).

**Fig. 4:**
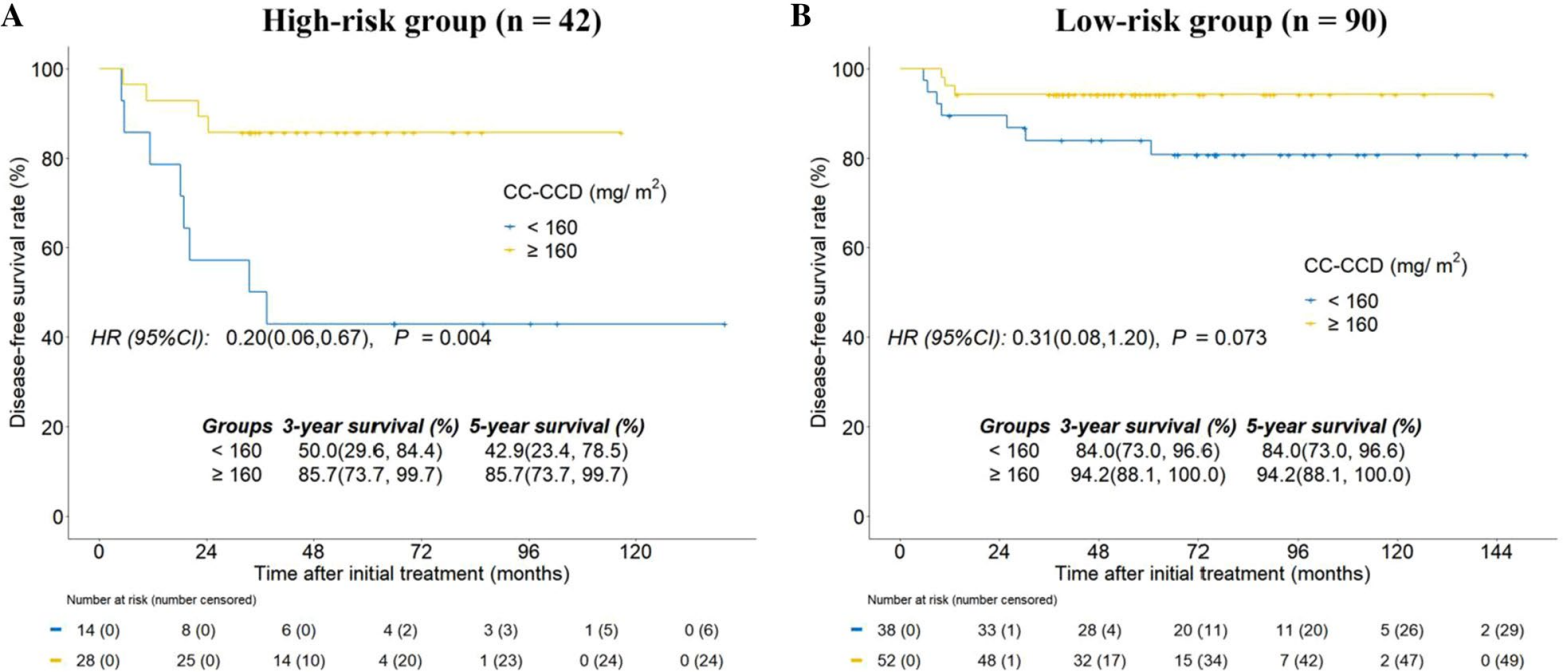
Kaplan–Meier curves of disease-free survival for the high-risk group (**A**) and low-risk group (**B**) stratified on CC-CCD levels (< 160 mg/m^2^ vs. ≥ 160 mg/m^2^). *CC-CCD* cumulative cisplatin dose during concurrent chemoradiotherapy

The low-risk group showed significantly improved 5-year DMFS (92.2% vs. 78.1%; P = 0.039; Fig. 3B) and OS rates (95.4% vs. 75.7%; P = 0.004; Fig. 3C) compared with the high-risk group. But no statistical variation was observed when comparing 5-year LRFS in the low-risk group versus the high-risk group (98.9% vs. 93.9%; P = 0.280; Fig. 3D). Subgroup analyses were performed in various risk groups based on RPA models to assess the survival benefit of CC-CCD (Fig. 4). In the high-risk group, participants who received CC-CCD ≥ 160 mg/m^2^ were seen to have significantly higher 5-year DFS rates than participants who received CC-CCD < 160 mg/m^2^ (85.7% vs. 42.9%; P = 0.004; Fig. 4A). Although participants who received CC-CCD ≥ 160 mg/m^2^ were more inclined to have superior 5-year DFS (94.2% vs. 84.0%; P = 0.073; Fig. 4B) in the low-risk group, this did not reach statistical significance.

### Severe acute and late toxicities

During chemoradiotherapy, severe (grade 3–4) mucositis was observed in 43.2% (57/132) of patients, and severe nausea was noted in 17.4% (23/132) of patients. Severe hematological toxicities included leucopenia (35/132; 26.5%), followed by neutropenia (18/132; 13.6%), anemia (5/132; 3.8%), and thrombocytopenia (2/132; 1.5%) during concurrent chemotherapy. During the follow-up, 115 (87%; 115/132) patients’ late toxicities were available. Severe late effects mainly included xerostomia (9.6%; 11/115), ototoxicity (7.8%; 9/115), neck fibrosis (5.2%; 6/115) and hypothyroidism (2.6%; 3/115). Overall, the rates of acute and late toxicities were comparable between high and low CC-CCD group (P > 0.05 for all; Table 3).

**Table 3:** Severe (grade 3–4) acute toxicity during concurrent chemotherapy and late toxicity during follow up

Adverse event	Entire cohort (n, %)	CC-CCD group (n, %)		*P* value^a^
		< 160 mg/m^2^	≥ 160 mg/m^2^	
Acute toxicity (n = 132)		n = 52	n = 80	
Mucositis	57 (43.2)	21 (40.4)	36 (45.0)	0.719
Nausea	23 (17.4)	10 (19.2)	13 (16.3)	0.648
Leucopenia	35 (26.5)	14 (26.9)	21 (26.3)	0.996
Neutropenia	18 (13.6)	9 (17.3)	9 (11.3)	0.437
Anemia	5 (3.8)	1 (1.9)	4 (5.0)	0.648
Thrombocytopenia	2 (1.5)	1 (1.9)	1 (1.3)	0.997
Late toxicity (n = 115)		n = 43	n = 72	
Xerostomia	11 (9.6)	5 (11.6)	6 (8.3)	0.723
Ototoxicity	9 (7.8)	4 (9.3)	5 (6.9)	0.726
Neck fibrosis	6 (5.2)	2 (4.7)	4 (5.6)	0.992
Hypothyroidism	3 (2.6)	2 (4.7)	1 (1.4)	0.555

*CC-CCD* cumulative cisplatin dose during concurrent chemoradiotherapy

^a^*P* values were calculated using the chi-square test or Fisher exact test if indicated

## Discussion

Based on our understanding, this is one of the first studies to assess the prognostic value of CC-CCD for CA-LANPC treated with NAC plus CCRT, especially for those patients who received favorable responses after NAC. Our results suggested that treatment with NAC and CCRT resulted in excellent outcomes (5-year OS, 89.3%) among CA-LANPC with favorable responses to NAC. Patients treated with CC-CCD < 160 mg/m^2^ indicated statistically diminished 5-year DFS compared to participants receiving CC-CCD of 160–200 mg/m^2^. However, no significant differences were observed between CC-CCD > 200 mg/m^2^ and 160–200 mg/m^2^ for DFS. Further analysis revealed CC-CCD ≥ 160 mg/m^2^ was associated with better DFS in high-risk patients among CA-LANPC; however, the benefit of CC-CCD ≥ 160 mg/m^2^ in low-risk patients requires further study.

The combination of NAC and CCRT has been studied on adults in multiple randomized clinical trials [21, 22]. An updated Meta-Analysis from the Nasopharyngeal Collaborative Group indicated that NAC plus CCRT was superior to just CCRT for disease control, locoregional control, and distant control in patients with LANPC [10]. However, randomized clinical trials have yet to assess childhood NPC, and high-level evidence on the treatment of children’s NPC is minimal. Over the past ten years, several small cohort studies have reported the survival outcomes of CA-LANPC after treatment with NAC plus CCRT. Overall, the 5-year DFS and DMFS rates varied from 56 to 79% and from 78 to 86%, respectively [23–26]. In the current study, treatment with NAC and CCRT showed great outcomes among CA-LANPC, with 5-year DFS rate of 83.9% and DMFS rate of 87.7%, respectively. In line with our results, a recent ARAR0331 study [27] found that the 5-year DFS rate and DMFS rate estimates were 84.3% and 89.2%, respectively. These data suggest that NAC combined with CCRT is a good treatment in the management of CA-LANPC.

An important note to address is that multiple NAC regimens (e.g. TP, PF, TPF, and GP) were adopted in the present study, which may confound the survival outcomes. However, the ideal NAC regimen was not established during the study period. Although the high-intensity TPF regimen appears to confer more benefits than the TP or PF combinations [8, 9, 28], statistically significant survival differences between neoadjuvant regimens were not detected by pooled analysis [22]. Neoadjuvant GP regimen is commonly used in most adult centers based on the phase III trial results [16, 29]. However, only four patients (3%) used the GP regimen in our study, because the GP regimen was still in the preliminary stage of NPC research during the study period. Also, there was insufficient clinical evidence to support its widespread use at the time. It is widely acknowledged that NAC has the advantages of micro-metastases eradication [10]. However, NAC may prolong the duration of treatment and reduce the compliance in subsequent CCRT [10]. The randomized controlled trials usually used two to three cycles of NAC before CCRT [8, 28, 30], but it remains unclear how many NAC cycles would be beneficial for patients. In our center, the idea of using NAC is mainly based on the response of NAC and the experience of clinicians. Once patients obtained CR/PR after NAC, the RT intervention would be considered as soon as possible to avoid RT delay and reduce the effect of treatment outcomes. Overall, all NAC regimens included in our study were consistent with the NCCN Guidelines [3], and the optimal NAC cycles remained unclear during the study period. To reduce the influence of the above factors on the results of the study, NAC-CCD, NAC cycles, and NAC regimens were all analyzed in the multivariate Cox proportional hazards models.

Patients who experienced favorable response after NAC had better treatment outcomes compared with patients who experienced unfavorable response after NAC [11, 12]. It can be speculated that the optimal CC-CCD used during RT may be different for patients with alternative responses after NAC. And the results of optimal CC-CCD would be affected if the NAC response was not considered. Recently, Liu et al. [31] attempted to determine an optimal level of CC-CCD in CCRT for NPC patients who completed CR/PR after NAC. They found participants receiving > 200 mg/m^2^ of CC-CCD after achieving CR/PR post NAC showed improved 3-year DFS and DMFS rates over participants receiving CC-CCD < 100 mg/m^2^. However, no significant distinctions were found between > 200 mg/m^2^ and 101–200 mg/m^2^ for all outcomes. Yet, Lv et al. [5] reported 200 mg/m^2^ of CC-CCD did not improve survival in LANPC, and 160 mg/m^2^ of CC-CCD may be sufficient for LANPC patients treated with NAC combined with CCRT. In the current study, the survival outcomes among CC-CCD < 160 mg/m^2^, 160–200 mg/m^2^, and > 200 mg/m^2^ were compared in CA-LANPC. Our data indicated that CC-CCD ≥ 160 mg/m^2^ was an independent protective prognostic factor for superior survival outcomes. However, no significant improvements for survival were seen in participants receiving > 200 mg/m^2^ of CC-CCD compared to participants receiving 160–200 mg/m^2^ of CC-CCD. This is in accordance with the study reported by Lv and his colleagues [5].

To confirm which patients benefit from high CC-CCD, previous studies usually use multivariate Cox regression analysis to elucidate all relevant prognostic factors [4, 5, 31]. However, these prognostic factors are often combined with other independent factors. To avoid the interaction between these factors and combine prognostic factors with risk of disease progression, all relevant risk factors were included in the RPA model for risk stratification analysis. We then analyzed the role of CC-CCD by hierarchical analysis through RPA, which stratified participants into subgroups with similar survival patterns. As a result, the RPA model then categorized CA-LANPC into different risk groups based on risk factors. In further stratified analysis, we found that the benefit of high CC-CCD is more obvious in the high-risk group. For the low-risk group, participants who received high CC-CCD were seen as having a distinct trend for superior 5-year DFS (94.2% vs. 84.0%; P = 0.073; Fig. 4B), but it was not statistically significant. Although the difference was non-significant in the low-risk group, they might also benefit from CC-CCD ≥ 160 mg/m^2^. Overall, larger cases and longer follow-up time are required for further study on the benefit of high CC-CCD for the low-risk group.

It is important to mention, most studies acknowledge T1/T2 and N0–1 as the early stages of cancer [32]. In this study, however, only 5 patients (3.8%) were staged at T1-2 and 19 (14.4%) were staged at N0–1 since the main focus was on the LANPC (stage III–IVB). Therefore, we combined T1–3 and N0–2 due to the limited number of patients with T1–2 and N0–1, respectively. Our findings suggested T stage was an independent prognostic factor in multivariate analysis. However, we failed to observe significance associated with N stage in the present study. This observation may be due to the limited sample size. In addition, nearly 80% of patients staged at N2 were in the N0–2 group. This overlap may make it more difficult to recognize the survival difference between N0–2 and N3 disease.

In adult patients with NPC, the overall reported response rate of NAC ranged from 67 to 77% [11, 32]. The response rate after NAC for two to four cycles was 90% in our study, which is higher than most of the other studies in adult patients. Consistent with our study [33], another prospective study (NPC-91-GPOH) reported the overall response rate to three courses of NAC (methotrexate, cisplatin, and 5-fluorouracil) was 91% in NPC children and adolescents. It’s important to note that more than half of patients received an NAC with TPF regimen, and approximately 40% received NAC with the TP/PF regimen in the current study, which are the most common and effective NAC regimens used in other series [18–22]. Consequently, it is reasonable to speculate that childhood and adolescent NPC has increased sensitivity to NAC compared to adult patients. However, latent mechanisms leading to the differences in NAC sensitivity between children and adults needs further study.

We should note our study has important limitations. Foremost, all utilized data was provided by one center in an NPC endemic area, thus reducing the ability to externally validate with data from other hospitals. Thus, our findings still need to be further validated. Next, limited by the small sample size of SD/PD patients after NAC (n = 15) in our study, the optimal CC-CCD for SD/PD patients was lacking. However, NPC in children and adolescents is rare, and identifying more may be a challenge since only 1–2% of all diagnosed cases of NPC were classified as childhood NPC in the endemic region. Finally, selection bias was an inevitability given the retrospective nature of this study. However, hierarchical analysis was employed to analyze the impact of CC-CCD in different risk patients, which is helpful to reduce the influence of confounding factors on the study results.

## Conclusions

In conclusion, CA-LANPC patients who received CC-CCD ≥ 160 mg/m^2^ showed superior DFS and DMFS rates compared to patients receiving CC-CCD < 160 mg/m^2^ while no significant variation was noted when comparing patients in the 160–200 mg/m^2^ and > 200 mg/m^2^ groups. Furthermore, we found that the benefit of CC-CCD ≥ 160 mg/m^2^ is more obvious in the high-risk group, but whether the low-risk group could benefit from CC-CCD ≥ 160 mg/m^2^ requires further study. Our results may contribute to improved guidance on individualized treatment strategies for CA-LANPC, especially for those patients who achieved CR/PR after NAC. However, a prospective study is still needed to verify our findings in the future.

## Supplementary Information

**Additional file 1: Table S1.** Baseline characteristics of childhood nasopharyngeal carcinoma stratified by CC-CCD (< 160 mg/m^2^ vs. 160–200 mg/m^2^ vs. > 200 mg/m^2^).

**Additional file 2: Figure S1.** Kaplan–Meier survival curves for disease-free survival stratified by CC-CCD levels (< 160 mg/m^2^ vs. 160–200 mg/m^2^ vs. > 200 mg/m^2^). CC-CCD, cumulative cisplatin dose during concurrent chemoradiotherapy.

**Additional file 3: Figure S2.** Kaplan–Meier’s disease-free survival (A), distant metastasis-free survival (B), overall survival (C) and locoregional relapse-free survival (D) curves for the entire group stratified by CC-CCD levels (< 160 mg/m^2^ vs. ≥ 160 mg/m^2^). CC-CCD, cumulative cisplatin dose during concurrent chemoradiotherapy.

**Additional file 4: Figure S3.** Kaplan–Meier’s disease-free survival (A), distant metastasis-free survival (B), overall survival (C) and locoregional relapse-free survival (D) curves for the entire group stratified by NAC-CCD levels (< 180 mg/m^2^ vs. ≥ 180 mg/m^2^). NAC-CCD, cumulative cisplatin dose during neoadjuvant chemotherapy.

**Additional file 5: Figure S4.** Kaplan–Meier survival curves for disease-free survival stratified on T stage and EBV DNA in all patients (n = 132). Class 1: T1–3 disease with EBV DNA < 4000 copy/mL; Class 2: T1–3 disease with EBV DNA ≥ 4000 copy/mL; Class 3: T4 disease with EBV DNA < 4000 copy/mL; Class 4: T4 disease with EBV DNA ≥ 4000 copy/mL.

## Data Availability

Key raw data were uploaded onto the Research Data Deposit public platform (RDD), with the approval RDD number of RDDA2021230562.

## Abbreviations

NPC: Nasopharyngeal carcinoma
NCCN: National Comprehensive Cancer Network
LANPC: Locoregionally advanced nasopharyngeal carcinoma
NAC: Neoadjuvant chemotherapy
CCRT: Concurrent chemoradiotherapy
CA-LANPC: Children and adolescents with locoregionally advanced nasopharyngeal carcinoma
RT: Radiotherapy
CC-CCD: Cumulative cisplatin dose during concurrent chemoradiotherapy
AJCC: American joint Committee on cancer
CR: Complete response
PR: Partial response
SD: Stable disease
PD: Disease progression
DFS: Disease-free survival
IMRT: Intensity-modulated radiotherapy
MRI: Magnetic resonance imaging
CT: Computed tomography
PET: Positron emission tomography
EBV: Epstein–Barr virus
PF: Cisplatin plus 5-fluorouracil
TP: Docetaxel plus cisplatin
TPF: Docetaxel, cisplatin, and 5-fluorouracil
GP: Gemcitabine plus cisplatin
RECIST: Response Evaluation Criteria in Solid Tumors
OS: Overall survival
DMFS: Distant metastasis-free survival
LRFS: Locoregional relapse-free survival
RPA: Recursive partitioning analysis
BMI: Body mass index
NAC-CCD: Cumulative cisplatin dose during neoadjuvant chemotherapy
