# Supplementary material for: Impact of cumulative cisplatin dose in childhood nasopharyngeal carcinoma based on neoadjuvant chemotherapy response in the intensity-modulated radiotherapy era: a real-world study
Source: Cancer Cell Int. 2021 Nov 12;21:604. doi: 10.1186/s12935-021-02281-4 (PMC8588629; doi:10.1186/s12935-021-02281-4)
Supplement: Supplementary file 1 — Additional file 1: Table S1. Baseline characteristics of childhood nasopharyngeal carcinoma stratified by CC-CCD (< 160 mg/m2 vs. 160–200 mg/m2 vs. > 200 mg/m2). [file 12935_2021_2281_MOESM1_ESM.docx]

**Table S1.** Baseline characteristics of childhood nasopharyngeal carcinoma stratified by CC-CCD (< 160 mg/m^2^ vs 160-200 mg/m^2^ vs > 200 mg/m^2^).

|  | CC-CCD group, mg/m^2^ | | |  |
| --- | --- | --- | --- | --- |
| Characteristic | < 160 (n = 52, %) | 160-200 (n = 50, %) | > 200 (n = 30, %) | *P* value^a^ |
| Sex |  |  |  | 0.331 |
| Male | 39 (75.0) | 31 (62.0) | 22 (73.3) |  |
| Female | 13 (25.0) | 19 (38.0) | 8 (26.7) |  |
| Age, years |  |  |  | 0.624 |
| ≤ 15 | 25 (48.1) | 29 (58.0) | 15 (50.0) |  |
| < 15 | 27 (51.9) | 21 (42.0) | 15 (50.0) |  |
| Smoking history |  |  |  | 0.073 |
| No | 46 (88.5) | 50 (100.0) | 27 (90.0) |  |
| Yes | 6 (11.5) | 0 (0.0) | 3 (10.0) |  |
| BMI, m^2^ |  |  |  | 0.421 |
| ≤ 1.45 | 22 (42.3) | 28 (56.0) | 14 (46.7) |  |
| > 1.45 | 30 (57.7) | 22 (44.0) | 16 (53.3) |  |
| EBV DNA, copy/mL |  |  |  | 0.168 |
| < 4000 | 17 (32.7) | 25 (50.0) | 10 (33.3) |  |
| ≥ 4000 | 35 (67.3) | 25 (50.0) | 20 (66.7) |  |
| T stage ^b^ |  |  |  | 0.409 |
| T1-3 | 29 (55.8) | 21 (42.0) | 14 (46.7) |  |
| T4 | 23 (44.2) | 29 (58.0) | 16 (53.3) |  |
| N stage ^b^ |  |  |  | 0.170 |
| N0-2 | 38 (73.1) | 30 (60.0) | 16 (53.3) |  |
| N3 | 14 (26.9) | 20 (40.0) | 14 (46.7) |  |
| Overall stage ^b^ |  |  |  | 0.277 |
| III | 19 (36.5) | 11 (22.0) | 6 (20.0) |  |
| IVA | 19 (36.5) | 19 (38.0) | 10 (33.3) |  |
| IVB | 14 (26.9) | 20 (40.0) | 14 (46.7) |  |
| NAC cycles |  |  |  | 0.087 |
| 2 | 30 (57.7) | 22 (44.0) | 8 (26.7) |  |
| 3 | 21 (40.4) | 25 (50.0) | 20 (66.7) |  |
| 4 | 1 (1.9) | 3 (6.0) | 2 (6.7) |  |
| NAC regimens |  |  |  | <0.001 |
| TPF | 18 (34.6) | 33 (66.0) | 22 (73.3) |  |
| TP | 13 (25.0) | 4 (8.0) | 3 (10.0) |  |
| PF | 21 (40.4) | 12 (24.0) | 2 (6.7) |  |
| GP | 0 (0.0) | 1 (2.0) | 3 (10.0) |  |
| Adjuvant chemotherapy |  |  |  | 0.460 |
| No | 50 (96.1) | 50 (100.0) | 29 (96.7) |  |
| Yes | 2 (3.9) | 0 (0.0) | 1 (3.3) |  |
| NAC-CCD, mg/m^2^ |  |  |  | <0.001 |
| < 180 | 34 (65.4) | 22 (44.0) | 6 (20.0) |  |
| ≥ 180 | 18 (34.6) | 28 (56.0) | 24 (80.0) |  |
| Radiation dose, cGy |  |  |  | 0.243 |
| < 6800 | 22 (44.0) | 23 (46.9) | 19 (63.3) |  |
| ≥ 6800 | 28 (56.0) | 26 (53.1) | 11 (36.7) |  |

**Abbreviations:** CC-CCD, cumulative cisplatin dose during concurrent chemoradiotherapy; BMI, body mass index; EBV, Epstein-Barr virus; NAC, neoadjuvant chemotherapy; TPF, cisplatin with 5-fluorouracil and toxoids; TP, cisplatin with toxoids; PF, cisplatin with 5-fluorouracil; GP, cisplatin with gemcitabine; NAC-CCD, cumulative cisplatin dose during neoadjuvant chemotherapy.

^a^*P* values were calculated using the chi-square test or Fisher exact test if indicated

^b^According to the 7^th^ edition of the Union for International Cancer Control/ American Joint Committee on Cancer staging system.
