# Supplementary figures and images for: Impact of cumulative cisplatin dose in childhood nasopharyngeal carcinoma based on neoadjuvant chemotherapy response in the intensity-modulated radiotherapy era: a real-world study
Source: Cancer Cell Int. 2021 Nov 12;21:604. doi: 10.1186/s12935-021-02281-4 (PMC8588629; doi:10.1186/s12935-021-02281-4)

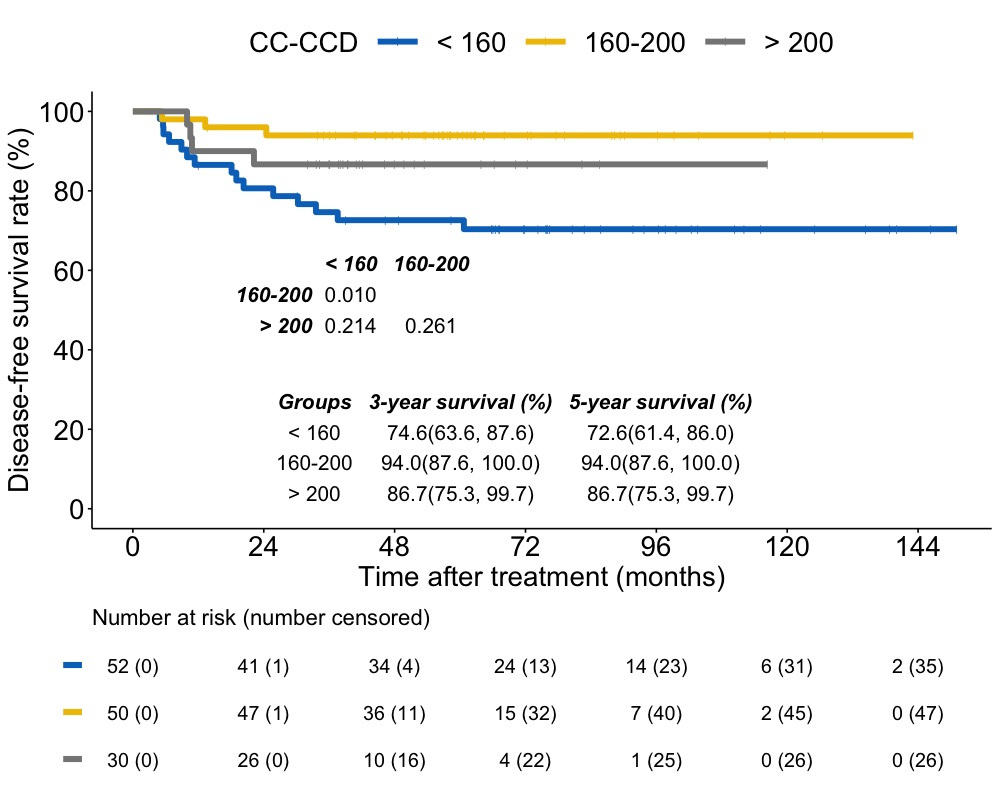

Supplement: Supplementary file 2 — Additional file 2: Figure S1. Kaplan–Meier survival curves for disease-free survival stratified by CC-CCD levels (< 160 mg/m2 vs. 160–200 mg/m2 vs. > 200 mg/m2). CC-CCD, cumulative cisplatin dose during concurrent chemoradiotherapy. [file 12935_2021_2281_MOESM2_ESM.jpg]

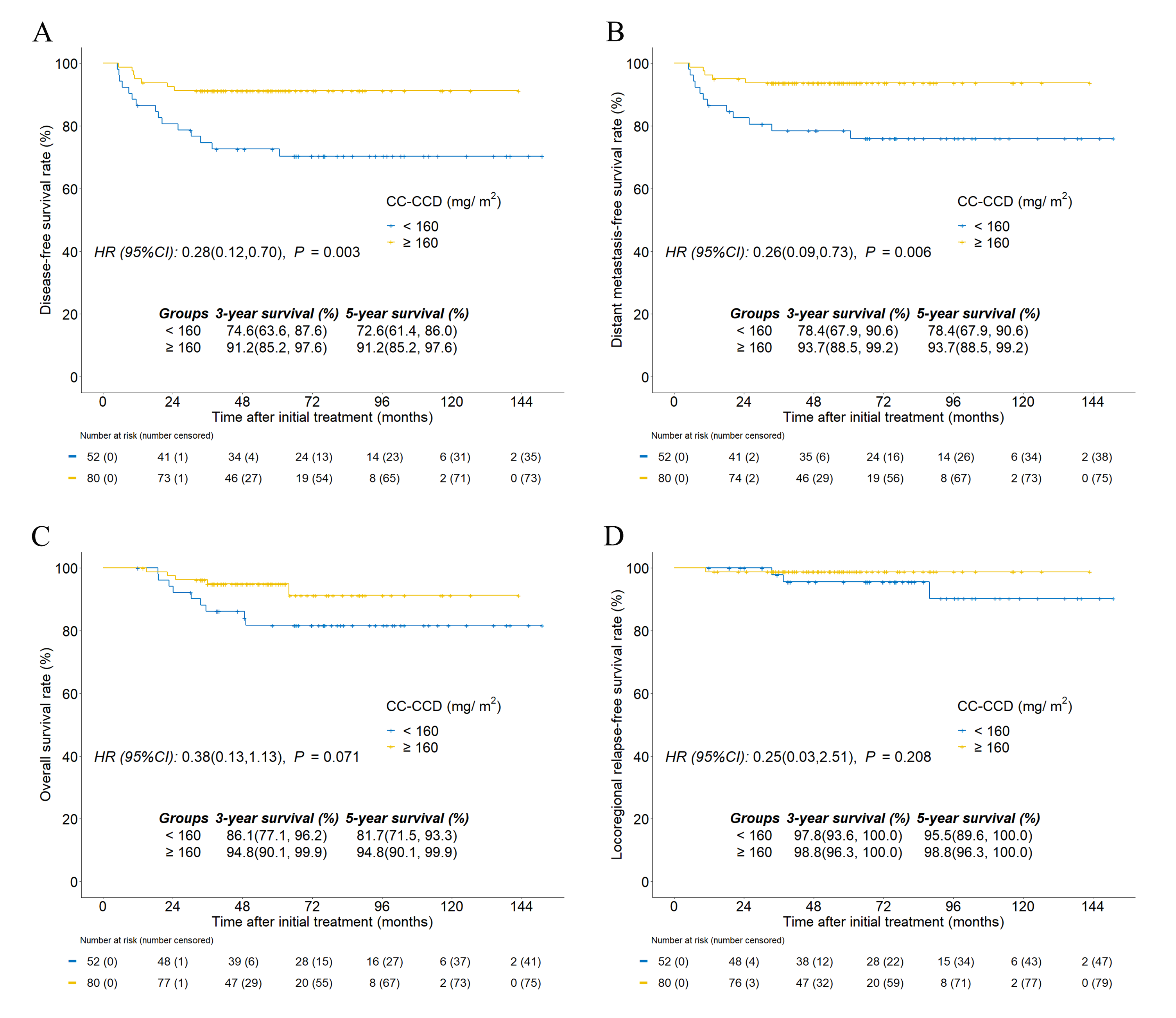

Supplement: Supplementary file 3 — Additional file 3: Figure S2. Kaplan–Meier’s disease-free survival (A), distant metastasis-free survival (B), overall survival (C) and locoregional relapse-free survival (D) curves for the entire group stratified by CC-CCD levels (< 160 mg/m2 vs. ≥ 160 mg/m2). CC-CCD, cumulative cisplatin dose during concurrent chemoradiotherapy. [file 12935_2021_2281_MOESM3_ESM.tif]

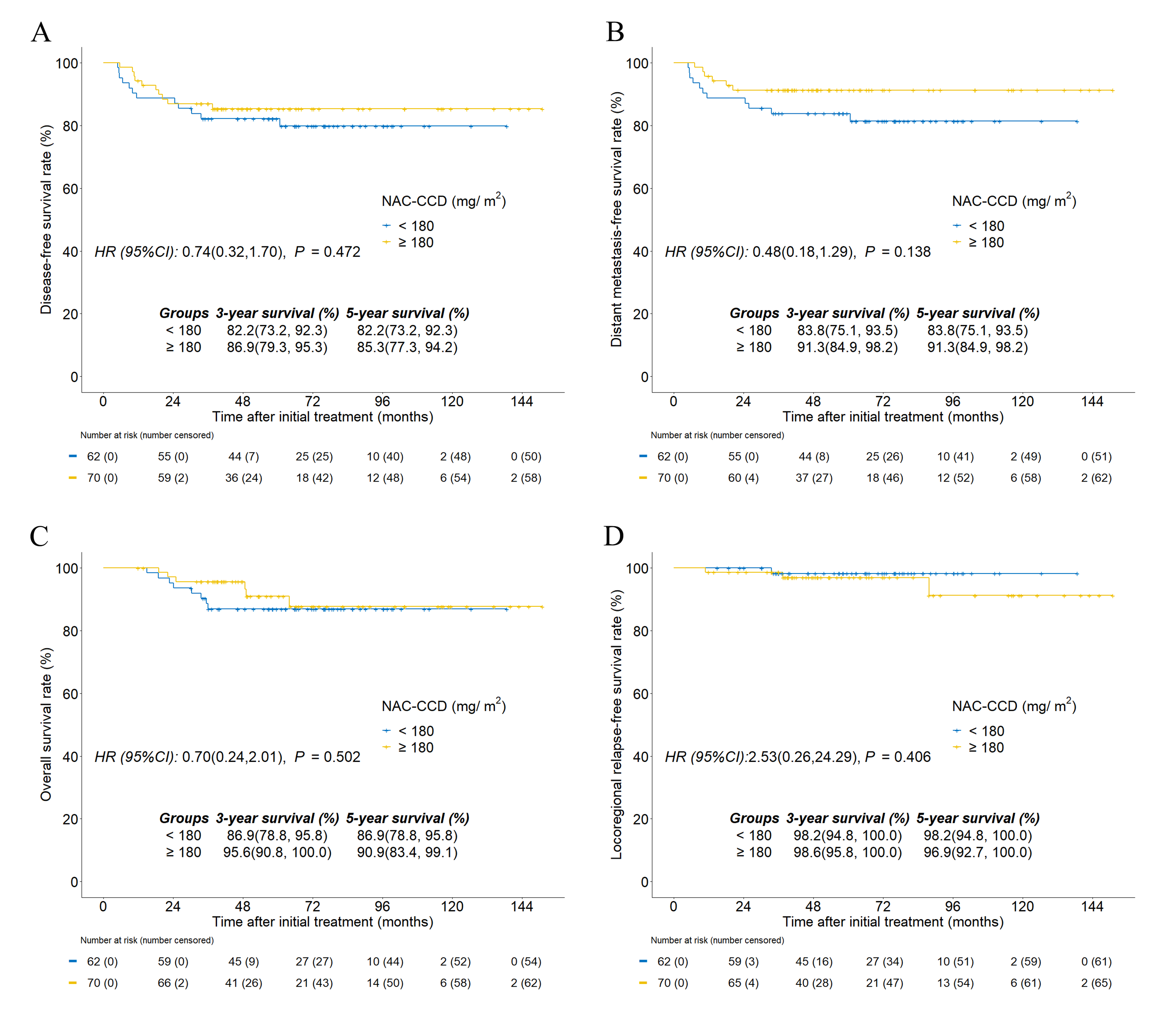

Supplement: Supplementary file 4 — Additional file 4: Figure S3. Kaplan–Meier’s disease-free survival (A), distant metastasis-free survival (B), overall survival (C) and locoregional relapse-free survival (D) curves for the entire group stratified by NAC-CCD levels (< 180 mg/m2 vs. ≥ 180 mg/m2). NAC-CCD, cumulative cisplatin dose during neoadjuvant chemotherapy. [file 12935_2021_2281_MOESM4_ESM.tif]

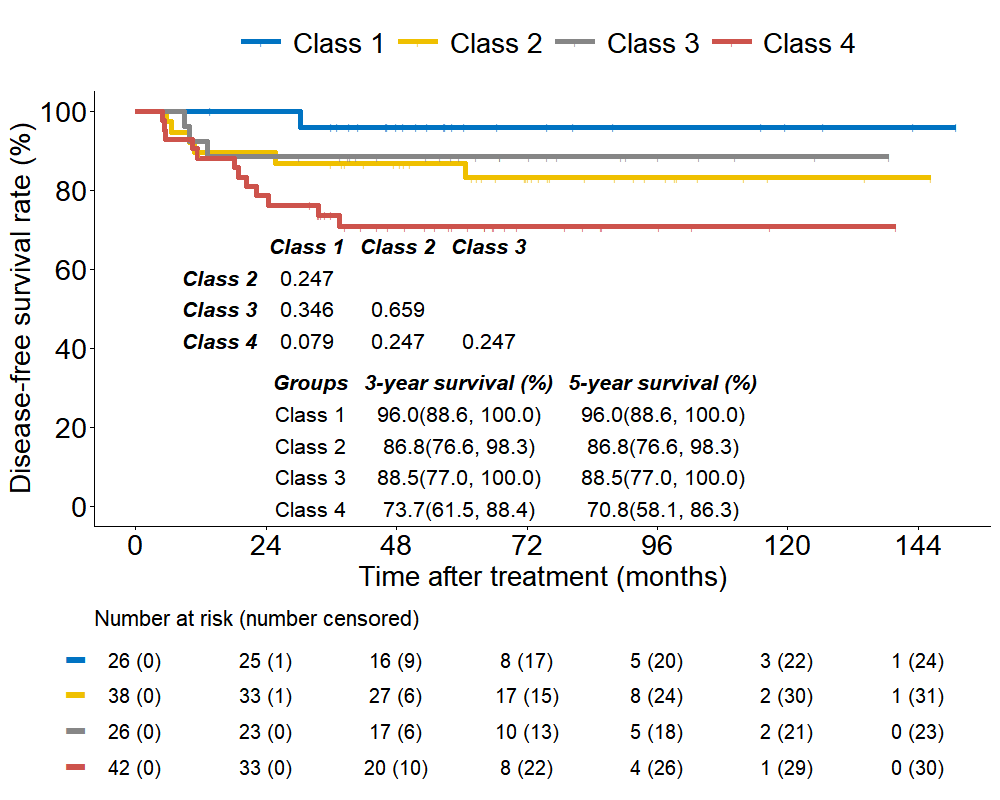

Supplement: Supplementary file 5 — Additional file 5: Figure S4. Kaplan–Meier survival curves for disease-free survival stratified on T stage and EBV DNA in all patients (n = 132). Class 1: T1–3 disease with EBV DNA < 4000 copy/mL; Class 2: T1–3 disease with EBV DNA ≥ 4000 copy/mL; Class 3: T4 disease with EBV DNA < 4000 copy/mL; Class 4: T4 disease with EBV DNA ≥ 4000 copy/mL. [file 12935_2021_2281_MOESM5_ESM.tiff]
